# Simulation theory of mind for heterogeneous human-robot teams

**DOI:** 10.3389/frobt.2025.1533054

**Published:** 2025-06-17

**Authors:** Monica Nicolescu, Janelle Blankenburg, Bashira Akter Anima, Mariya Zagainova, Pourya Hoseini, Mircea Nicolescu, David Feil-Seifer

**Affiliations:** Robotics Research Laboratory, Department of Computer Science and Engineering, University of Nevada, Reno, NV, United States

**Keywords:** theory of mind, robot teams, task allocation, heterogeneous robots, distributed control

## Abstract

This paper focuses on the problem of collaborative task execution by teams comprising of people and multiple heterogeneous robots. In particular, the problem is motivated by the need for the team members to dynamically coordinate their execution, in order to avoid overlapping actions (i.e. multiple team members working on the same part of the task) and to ensure a correct execution of the task. This paper expands on our own prior work on collaborative task execution by single human-robot and single robot-robot teams, by taking an approach inspired by simulation Theory of Mind (ToM) to develop a real-time distributed architecture that enables collaborative execution of tasks with hierarchical representations and multiple types of execution constraints by teams of people and multiple robots with variable heterogeneity. First, the architecture presents a novel approach for concurrent coordination of task execution with both human and robot teammates. Second, a novel pipeline is developed in order to handle automatic grasping of objects with unknown initial locations. Furthermore, the architecture relies on a novel continuous-valued metric which accounts for a robot’s capability to perform tasks during the dynamic, on-line task allocation process. To assess the proposed approach, the architecture is validated with: 1) a heterogeneous team of two humanoid robots and 2) a heterogeneous team of one human and two humanoid robots, performing a household task in different environmental conditions. The results support the proposed approach, as different environmental conditions result in different and continuously changing values for the robots’ task execution abilities. Thus, the proposed architecture enables adaptive, real-time collaborative task execution through dynamic task allocation by a heterogeneous human-robot team, for tasks with hierarchical representations and multiple types of constraints.

## 1 Introduction

Current techniques for human-robot teamwork focus on supervisory control of (semi-) autonomous or teleoperated robotic systems by human operators. Although advances have been made in the area of collaborative robots (co-bots), numerous challenges remain for embedding human agents as teammates with an autonomous robot team. A central problem for task execution in human-robot teams is the coordination of actions between teammates that have different communication mechanisms and representations, to ensure that there is no overlap between their actions and that the task is properly executed according to its constraints. The task allocation and team-self organization is further challenged by the heterogeneity and variability in robot performance during a task.

**FIGURE 1:**
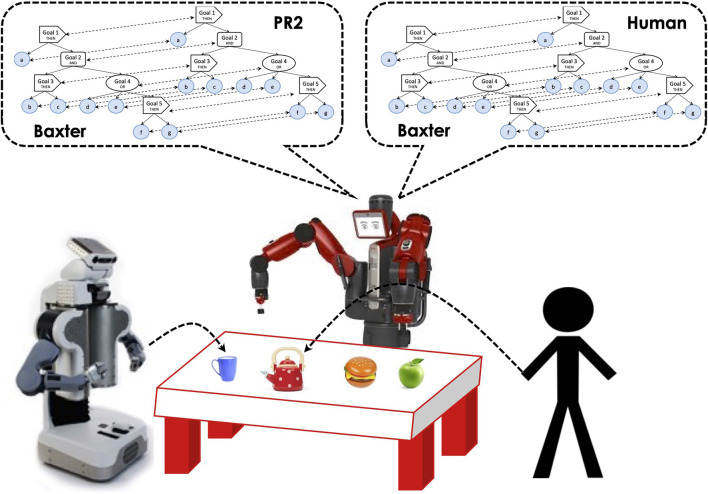
Human-robot teamwork using a ToM approach, from the perspective of the Baxter robot.

We propose an approach in which each team member has its own copy of the task representation, as shown in Figure 1. For the robots, the representation is a hierarchically structured tree that incorporates all the tasks constraints and also acts as the controller that enables the robot to perform the task. For robot-robot coordination, explicit messages are passed between sibling nodes of the robots’ controllers. The human teammate also has knowledge of the task constraints, but their mental representation is unavailable to the robots. To enable coordination with people, in a similar way as the coordination with robot teammates, we propose a simulation Theory of Mind approach in which the robots store a simulated copy of the human’s task representation, which is continuously updated from visual observations to track the human’s current working goals as well as to record past goals achieved. This is described in detail in Section 2.3.

From the perspective of robot-only teams, a central problem is the allocation of robots to task(s). Finding an optimal solution to this problem is an instance of the MT-MR problem (Multi-Task robots performing Multi-Robot tasks) as defined in (Gerkey and Matarić, 2004). Heterogeneous robot teams have a more complex problem due to the different capabilities of the robots. While numerous approaches have been developed for both collaborative task execution and heterogeneous teams, this paper addresses these problems from a different perspective, with a focus on tasks with complex hierarchical constraints, in order to expand the capabilities of heterogeneous multi-robot systems.

In current approaches for multi-robot control, a robot’s ability to perform certain actions or tasks (i.e., the team’s heterogeneity) is known *a priori* and is represented by a binary value depicting whether the robot can or cannot perform a task (without any other options in between). These approaches also assume that these capabilities are not changing over time (i.e., the robot has a fixed set of skills throughout the entire task execution). While there are situations in which there is a clear binary choice on a particular capability (e.g., a robot without a mobile base cannot move), there are also many situations for which the degree to which a robot may perform a task covers a continuous spectrum. For instance, a robot with a dexterous hand may pick up objects better than a robot with a parallel gripper; both are able to perform the task, though with varying degrees of effectiveness. Having a continuous-valued metric that encodes this information can be highly beneficial for task allocation in a multi-robot team, as it would allow the selection of the robot best suited for a given task. This is especially important for hierarchical tasks in which portions of the task must be completed correctly before the next step can begin, such as assembly tasks. This metric would help to avoid issues with future steps in a hierarchical task which rely on proper completion of prior tasks by ensuring robots are allocated tasks which they can complete effectively.

Furthermore, the value of this metric may vary throughout the task execution, as different environmental conditions may change the degree to which the robot is able to perform the task or may allow it to perform actions that would otherwise be impossible. This paper introduces a metric that encodes a robot’s ability to perform a particular task component (grasping of objects) over both a continuous and discrete spectrum. The metric utilizes our novel perception-manipulation pipeline, which is able to automatically generate grasps for objects with unknown initial locations. The metric is updated continuously, allowing for dynamic task allocation that takes into account current environmental conditions.

The control architecture presented in this paper enables multiple heterogeneous robots to coordinate their task execution with a human. Furthermore, the architecture uses the novel metric, which incorporates a robot’s varying capabilities, to enable dynamic, real-time task allocation in the context of hierarchical tasks that have the following type of execution constraints: 1) sequential: all the steps must be performed in a fixed given order, 2) non-ordering: all the steps must be performed, but the order is irrelevant, and 3) alternative paths: either some or others of the steps may be performed in order to achieve the same goals. This work focuses on complex tasks with the assumption that all of these constraints may occur in the same task. Experiments with physical robots are performed with a heterogeneous team of two humanoid robots, with and without the metric, to evaluate the usefulness of incorporating information about heterogeneity in the task allocation process. A human is added to this team and experiments are performed to illustrate the ability of the robots to coordinate their task execution with each other as well as a human partner.

## 2 Materials and methods

### 2.1 Related work

The problem addressed in this paper falls in the areas of human-robot collaboration and multi-robot task allocation (MRTA) (Gerkey and Matarić, 2004). A collaborative robot should be able to execute complex tasks (Fraser et al., 2016), be aware of its teammates’ goals and intentions (Kelley et al., 2008), as well as be able to make decisions for its actions based on this information. In recent work Hawkins et al. (2013) use a probabilistic approach to predict human actions and employ a cost based planner to determine the robot response. Prediction of human actions is performed using a forward-backward message passing algorithm in a Bayes network representation of the task. However, a full conditional probability table is needed for inference, which increases the complexity of the process for large tasks. An extension of this work, with a new task representation that includes multiple paths of execution, has been presented in (Hawkins et al., 2014). The tasks are encoded as AND-OR tree structures that need to be converted to an equivalent Bayes network, with all alternative paths explicitly enumerated, in order to support action prediction and planning. The task tree representation used in this paper has a THEN-AND-OR tree structure which also allows for sequential constraints. Furthermore, the proposed approach enables a robot to make decisions based on a simulated mental model of the human’s task, without having to enumerate all possible alternative paths of execution. More recently, Heppner et al. (2024) introduce an approach in which robot capabilities are explicitly modeled as nodes in a behavior tree and a auction-based system enables run-time coordination between heterogeneous robots in a find-and-decontaminate mission. In our proposed work, the robots consider their own utility to achieving task goals, similarly to (Heppner et al., 2024), but combine this with mental models of the other agents in the team, including humans, in order to decide which goals to pursue. Fusaro et al. (2021) propose a reactive task planner that generates behavior trees that uses action-related costs and takes into account the presence of a human teammate. In our proposed work, all agents in the team have knowledge of the same task representation, while the self-organization of the team is achieved dynamically at run time, based on individual action cost assessment and knowledge of the mental models of the other teammates.

Effective teamwork relies on understanding of the teammates’ intentions, in order to properly decide how to allocate the task across the team. Intent recognition is a part of Theory of Mind (ToM), providing the ability to interpret and predict other’s behaviors. In the robotics domain, Kelley et al. (2008) present a Hidden Markov Model based system for detecting human navigation intentions using a simulation ToM approach (Gurney and Pynadath, 2022), in which a robot predicts human actions by taking their perspective and aiming to predict what it would do if it were performing the same actions. In another approach relying on simulation theory, Nguyen et al. (2018) propose a Dueling Deep Q-Network and LSTM action learning mechanism for generating an agent’s own decision-making model, which can later be used to predict the intentions of others in a block building task. The system requires the agents to take turns during collaborative execution, with one being observer while the other takes actions according to its goals and perceived intentions of the other. Hierarchical models of task representations have also been employed in conjunction with ToM approaches for intent recognition. Saffar et al. (2015) incorporates contextual information with an Activation Spreading Network, in order to enable a robot to disambiguate a human user’s intention and naturally interact during a complex household task. In (Gao et al., 2020) an And-Or hierarchical model of a collaborative task along with a Bayesian inference approach is used in order to build a mind model of a human user and to generate explanations about the task. Bayesian inference has also been used by (Hernandez-Cruz et al., 2024), in a multi-modal framework that fuses head/hand orientation and hand movement in order to detect a human’s grasp intent in a tabletop human-robot collaborative scenario. These methods, as well as our approach, focus on the specific aspect of modeling the intentions of others. While ToM includes other important aspects (such as mental states, beliefs, thoughts, emotions), which can enable more complex forms of reasoning, for the purpose of this work, we propose an approach that relies on the ability to recognize and represent others’ intentions in a simulated mental model of their task representations, in order to enable cooperative task execution in heterogeneous teams.

In the area of human-robot teamwork, existing approaches focus on optimizing the task allocation problem (Gombolay et al., 2018), which result in approaches that cast the robots into fixed roles that do not change over the course of the task execution. For example, Gombolay et al. (2015) assumes that both the robot and humans in the team have a fixed set of skills and full *a priori* knowledge of the task details. In addition, both the robot and humans have the capability of allocating sub-tasks to the individual teammates. Once the task allocation has been completed, the team members perform the task independently, with no interactions. In practical applications the agents may have to work jointly on parts of the task and their roles might change due to changes in the task at run-time. In this research, an architecture is developed to take into account the dynamic nature of teamwork and can handle real-time task allocation based on the current state of the environment.

Early implementations of multi-robot systems include actress (Asama et al., 1989), alliance (Parker, 1994), and murdoch (Gerkey and Matarić, 2000). We focus on 1) dynamic team heterogeneity in the context of 2) complex tasks with hierarchical representations and multiple types of execution constraints. To handle heterogeneity in multi-robot teams, Parker (1994) proposed an architecture called alliance and a related L-alliance architecture (Parker, 1996). These approaches use continuous valued metrics for heterogeneity, but the values stay fixed during the task and therefore cannot reflect environmental changes as our approach does. Werger and Matarić (2000) presents a distributed behavior based approach to the problem of Cooperative Multi-Robot Observation of Multiple Moving Targets (CMOMMT). The architecture uses cross inhibition and cross subsumption between peer behaviors on each robot to determine allocation of robots to targets, but all the robots have the same capabilities and thus does not consider any heterogeneity. Gerkey and Matarić (2002); Gerkey and Matarić (2000) proposed MURDOCH, a dynamic task allocation approach for a group of heterogeneous robots utilizing a publish/subscribe messaging system. The approach is also validated with both a tightly coupled multi-robot physical manipulation task and a loosely coupled multi-robot experiment with long-term autonomy that included tasks such as object-tracking, sentry-duty, cleanup, and monitor-object. Although the tasks were somewhat complex, their inherent constraints were mostly limited to sequential constraints between atomic behaviors. Our proposed approach allows for more complex constraints such as non-ordering and multiple paths of execution.

Additional market-based architectures focus on allocating tasks distributively (Dias et al., 2006), while the team seeks to optimize an objective function based upon individual robot utilities for performing particular tasks (Parker, 2008). Wang et al. (2016) proposed an algorithm which utilizes a task evaluation function based on distance fitness and urgency. CeCoTA is a market based algorithm for simultaneous allocation of multiple tightly couple multi-robot tasks to coalitions of heterogeneous robots (Das et al., 2014). The approach was validated in a simulated environment with robots allocating a set of atomic tasks. However, the work does not incorporate any inter-task dependencies, which are the basis of our hierarchical task constraints.

Coalition formation is a prevalent approach for handling team heterogeneity, enabling multiple robots to build small teams that allow them to perform a larger overall task. ASyMTRe enables the sharing of sensory and computational capabilities (Zhang and Parker, 2010) in a navigation task in which only one of the robots has localization capabilities. This approach was extended in (Zhang et al., 2014), demonstrating formation of coalitions in tightly coupled multi-robot tasks that need to maintain a set of given sensor constraints, in a domain in which robots need to navigate to various goals. Similar coalitions have been demonstrated in cooperative manipulation tasks: Chaimowicz et al. (2001) demonstrate an approach based on two-robot leader/follower coalitions to carry a box. Furthermore, Huntsberger et al. (2003), Huntsberger et al. (2004) present campout, a Control Architecture for Multi-robot Planetary Outposts validated on physical experiments of coordinated object transport and team cliff traverse. However, these types of coalition formation methods assume that the tasks are atomic behaviors which do not have any inter-task constraints. These inter-task constraints are the focus of our tasks which contain complex hierarchical constraints between the tasks.

Methods that aim to handle more complex task representations have been shown in (Koes et al., 2005; Ziparo et al., 2011), which focus on the execution of tightly coupled tasks. In (Koes et al., 2005), the task allocation problem is modeled as a mixed integer linear programming (MILP) problem and a centralized anytime algorithm is developed to provide an optimal solution that handles the allocation, scheduling and path planning for a search and rescue task with spatial constraints. Due to the centralized nature of the algorithm, the method is dependent on prior knowledge of a static environment and produces fixed allocations that do not change during the course of the task. The Petri Net Plan framework developed in (Ziparo et al., 2011) can represent multi-robot plans using sensing, loops, concurrency, non-instantaneous actions, action failures, and different types of action synchronization. This does not consider heterogeneity as a factor for task allocation. Furthermore, this method was tested on a homogeneous team of robots (AIBO’s) with equivalent capabilities.

The proposed metric is similar to the utility functions computed by the above market-based approaches and to the motivation factors used in the ALLIANCE architecture (Parker, 1994). It incorporates task specific utility (such as a distance to a target object) with both continuous utility (perceived grasp effectiveness) and discrete information about the robots’ skills (ability/inability to grasp a given object). However, the proposed work contributes this metric for hierarchically structured tasks, that exhibit a combination of complex constraints such as sequential, non-ordering, and alternative paths of execution by a team of heterogeneous robots.

### 2.2 Prior work on collaborative task execution

This work builds on two previously developed approaches for collaborative execution of complex tasks: 1) a distributed control architecture that allows for dynamic allocation of tasks in homogeneous robot teams (Blankenburg et al., 2017) and 2) a collaborative architecture that enables a single robot to coordinate its task with a single human user (Anima et al., 2019). This paper expands this work to teams comprising of heterogeneous robots and humans, bringing new challenges for coordination and communication.

**FIGURE 2:**
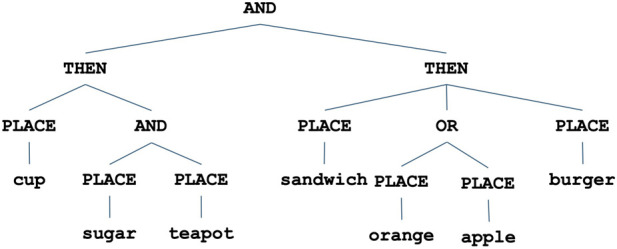
Representation of the joint task network for the heterogeneous team experiments.

The underlying architecture for single robot control (Fraser et al., 2016), which is the basis of this research, enables the encoding of hierarchical tasks involving constraints such as sequential (*THEN* nodes), non-ordering (*AND* nodes), and alternative paths of execution (*OR* nodes), as shown in Figure 2. This representation serves both as an encoding of the task constraints as well as the actual controller that is executed by the robot. *Goal Nodes* are the internal control nodes of the hierarchical task structure, and include the *THEN*, *AND*, and *OR* nodes that are used by the tree to encode the execution constraints of the task. *Behavior Nodes* are the leaf nodes in the task tree structure and encode the physical behaviors that the robot can perform, e.g., a behavior to pick and place a cup will control the arm of the robot to pick up a cup from the table and place it in another location. Each node in the architecture maintains a state consisting of several components: *activation level, activation potential, active*, and *done*. The state information is continuously maintained for each node and is used to perform top-down (*activation level*) and bottom-up (*activation potential*) activation spreading that ensures the proper execution of the task given the constraints. The state of each node in the task structure is maintained via an update loop which runs at each cycle. This loop uses the *activation potential* information to activate the node that has the most availability for action (e.g., highest potential).

In the *heterogeneous, multi-robot domain* Blankenburg et al. (2017), to enable cooperative execution of team tasks, each robot maintains its own instance of the joint task tree structure, identical to that of the other robots. Equivalent nodes in the task structures across robots are called *peers*. These peers are the means of communication between the robots and allow nodes to keep track of other robots’ progress on the task. While the task hierarchy is uniform across robots, the *activation potential* and *activation levels* for each node are calculated individually by each robot. Using the state of the peer nodes, each robot is able to identify if a given node is currently being worked on or was already completed by another robot. This is necessary to ensure there is no overlap in the sub-tasks that the robots perform. The activation spreading mechanism on a single robot utilizes the peer information in its own task tree to determine the next step it should perform. The process allows the robots to maintain and communicate the states of all of the nodes to their corresponding peer nodes on the other robots in order to ensure that the robots can work collaboratively to complete the task in a manner that follows its constraints (Fraser et al., 2016; Blankenburg et al., 2017).

In the *single human single robot domain* (Anima et al., 2019), the robot stores a simulated copy of the human’s task controller, representing the human’s mental model of the task. This second representation is kept in parallel with the robot’s own representation, and the status of various nodes in the human’s task (e.g., *working*, or *done*) is updated by the robot using its camera. Peer nodes on both the robot’s and the human’s controllers continuously exchange messages that communicate their status information, enabling the robot to infer what part of the task the human is working on. The robot decides its next action based both on the constraints of the joint task and the behavior of the human partner.

### 2.3 Simulation theory of mind approach to heterogeneous human-robot teams

Given a team of robots and a common task with hierarchical structure and temporal execution constraints the goal is to design an architecture that allows the team to dynamically self-organize (i.e., decide who does what), based on the current state of the environment. The major challenge in this context is to coordinate the actions of the teammates such that they do not overlap, meaning to work on the same parts of the task, and that the task is performed correctly according to the specified constraints. Our proposed solution is based on simulation theory, based on the underlying assumption that all the teammates have a complete and identical representation of the task constraints, allowing each of them to understand the states of the others from a similar perspective.

**FIGURE 3:**
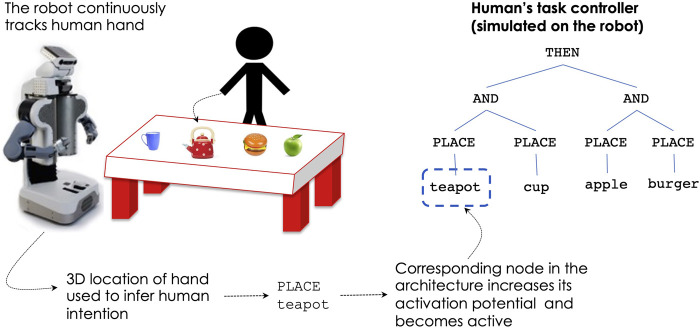
The human’s task controller is continuously updated based on detected human intent.

To enable coordination within the human-robot team, each robot teammate is equipped with several capabilities. First, each robot has its own task representation (in the form of a hierarchical tree as shown in Figure 2), which is the robot’s actual controller used to perform the task. All robots update their task controllers during execution, keeping track of which nodes are currently *active* and which are *done*. Beyond their own controller, each robot stores a separate mental model of the task representation for each of the human teammates. This takes the form of a simulated controller, identical in nature with the robot’s own, whose state is updated based on observations from the sensors, in order to keep track of the humans’ current and past goals achieved. The process is illustrated in Figure 3: the robot uses its sensors (depth camera, laser rangefinder) to track the location of the human’s hand in 3D coordinates. Based on the current and past hand locations, the robot infers what is the most likely object that the human is reaching for: from change in distance and vector movement of the hand toward all objects, the object of interest is the one for which the distance is decreasing the fastest and angle between the movement vector and hand vector to object is the smallest. As the object of interest (intent) is recognized, the robot increases the activation potential of the corresponding behavior node in the human’s task controller, causing the node to become active. The human’s task controller does not perform any actual work, it is only employed to keep an updated status of the human’s current goals and the parts of the task that have already been achieved. Thus, through this process, the visual tracking of the human’s hand, enables the robot to recognize the human’s intent in the context of tasks involving manipulation of objects. The robot ascribes the detected intent to the human, updating the corresponding mental model of the human’s task, which further enables the human-robot coordination, as described below.

In the proposed architecture, coordination is achieved through messages between peer nodes of the corresponding controllers, either from robot to robot or from robot to human controllers. Thus, during task execution, each robot controller continuously communicates with all other robot controllers as well as all the simulated human controllers, in order to ensure that the behavior it selects for execution obeys the task’s ordering constraints and that it is not in execution by another team member. When choosing a behavior to perform, there could be two situations: 1) none of the other teammates are currently working on, or have the intent to perform that behavior, and 2) another teammate is already working on or has the intention to perform that behavior. The communication and behavior activation process for these situations is described below, for both robot-robot and robot-human coordination.

**FIGURE 4:**
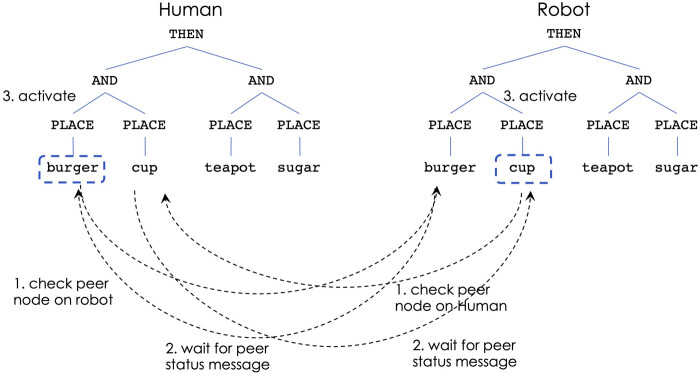
Human-robot decision making: non-overlapping sub-tasks.

**FIGURE 5:**
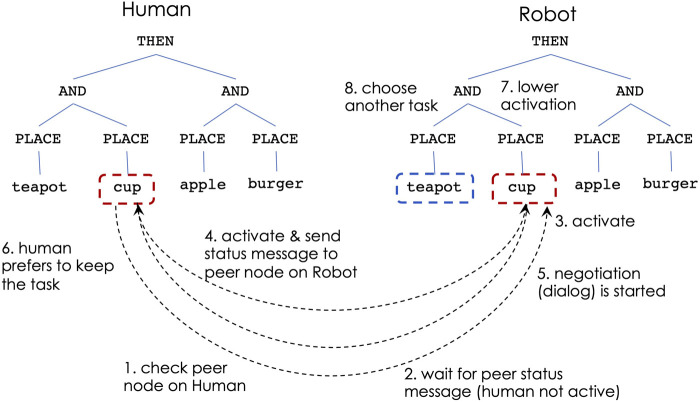
Human-robot decision making: overlapping sub-tasks, human prefers to continue

**FIGURE 6:**
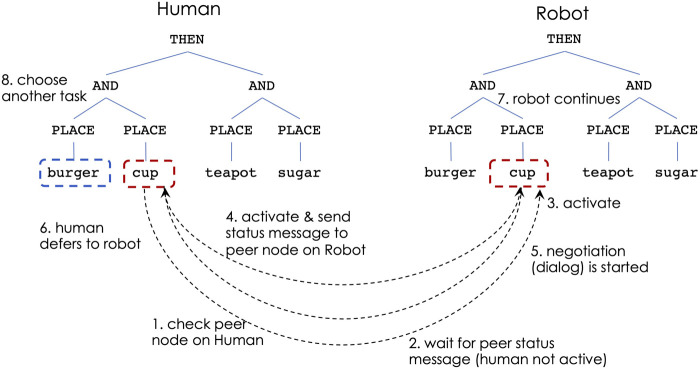
Human-robot decision making: overlapping sub-tasks, human concedes to robot.

Figures 4–6 show the steps of node activation for human-robot coordination, for a generic task of placing some food and kitchen items. Throughout the entire execution the robot continuously updates the human’s simulated controller in order to keep track with the human’s task execution.

Figure 4 illustrates the decision process for situations in which the human and robot choose to work on different sub-tasks. Due to the THEN constraint at the top of the task, the left sub-tree is the first that needs to be performed. The human decides to place the burger, which, through the robot’s continuous observations, leads to activating the PLACE-burger node from the human’s controller. At the same time, if the robot’s activation level is highest for PLACE-cup (due to closeness to that object), the robot aims to activate that behavior. At this time both PLACE-burger (from the human controller) and PLACE-cup (from the robot) send messages to check the peer nodes on the other’s controller (step 1). Next, the nodes receive peer status messages indicating that the other teammate does not work on the same sub-task (step 2). This leads to activating the corresponding behaviors on both controllers (step 3).

If the human and the robot decide to work on the same sub-task, a dialog is initiated by the robot to ask the human user if they prefer to continue (Figure 5) or if the task is conceded to the robot (Figure 6). The answers are given verbally with a yes/no, which is detected through a microphone. Of particular interest, are the situations when the human and robot decisions are made simultaneously, meaning that the human has initiated actions to the same sub-task as the robot, but the simulated controller model has not yet been updated to indicate it as an active sub-task. If the human’s peer subtask is active when the robot sends status check messages, the robot always concedes the subtask execution to the human.

Considering the same task as before, the behavior activation proceeds as follows: if the robot’s highest activation level is for PLACE-cup, the robot’s corresponding node will send a check status message to its peer from the simulated human controller. If the peer node responds as not active (step 2), the robot activates its PLACE-cup behavior (step 3). However, if the PLACE-cup node on human controller becomes active immediately after, a status message is sent from the human controller to the peer node on the robot (step 4). Since this represents a conflict of overlapping goals, the robot stops its execution and initiates a dialog (step 5). If the human indicates a preference to continue with the task (step 6) via a verbal response, the robot lowers its activation for the PLACE-cup behavior and chooses another task, which in this case is for PLACE-burger (step 8). If the human decides to concede the sub-task to the robot (step 6 in Figure 6), the robot continues with performing it (step 7) and the human chooses a different sub-task (step 8).

**FIGURE 7:**
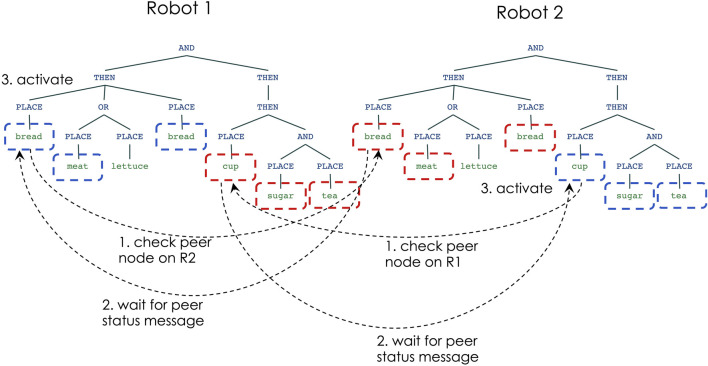
Multi-robot decision making: non-overlapping sub-tasks.

Figure 7 shows the steps of node activation for robot-robot coordination, in situations in which the robots choose to work on different sub-tasks: robot 1 begins by choosing to place the bread for the sandwich, while robot 2 begins by choosing to place the cup for the tea. Initially, the nodes for PLACE-bread (on robot 1) and PLACE-cup (on robot 2) check the status of the peer nodes on the other robot (step 1) and wait for the peer status message (step 2). Since the peer nodes indicate that the other robot does not intend to activate the same node, each robot decides that it can activate their nodes and begin the sub-task execution (step 3).

**FIGURE 8:**
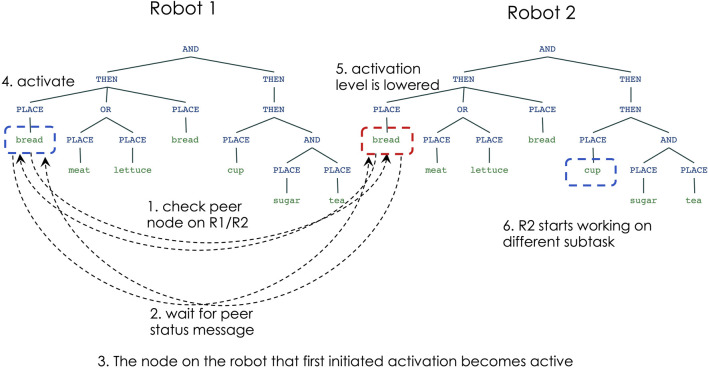
Multi-robot decision making: overlapping sub-tasks.

Figure 8 shows the node activation process when the robots decide to work on the same sub-task: in this scenario both robots choose to work on placing the bread for the sandwich. Initially (step 1) the nodes for PLACE-bread on both robots check the status of the peer nodes and then wait for their status message (step 2). The response messages indicate that both robots plan to work on the same node, but have a timestamp indicating which robot first initiated the activation. The robot that has the earliest activation timestamp would then activate its node (steps 3–4), while the other robot lowers its activation for the same sub-task (step 5). This enables another node in robot 2’s network (e.g., PLACE-cup) to get a higher activation level, and thus to begin working on another part of the task (step 6).

### 2.4 Task allocation for heterogeneous teams with dynamic capabilities

#### 2.4.1 Task allocation using activation potential

The architecture in Section 2.2 assumes that all the robots in the team have the same capabilities, all robots are capable of performing all the sub-tasks/behaviors, and the activation potential of each behavior node is computed using a distance-based metric. We introduce a generalized and extensible approach for considering the robot’s degree of ability to perform a task that: 1) covers a discrete and continuous spectrum and 2) is variable in different environmental conditions. This modulation of the architecture is able to handle cases where robots have different capabilities. This degree of ability can be utilized to compute the *activation potential* to reflect the dynamic grasp capabilities of the different robots.

To handle the variable heterogeneity between robots, several factors are incorporated into a single metric representing a robot’s perceived level of capability for executing a specific behavior. In addition, the metric is continuously updated during the task execution, enabling the team to take into account the most recent environmental conditions for task allocation. For the team of humanoid robots used for this work, the main type of behavior node used is a manipulation (pick and place) behavior (represented in short as PLACE in the task representations); therefore, the cues considered relevant for the metric are specific for manipulation tasks. The components taken into account are the distance between the arm and the objects and a grasp score that represents a robot’s perceived effectiveness of grasping an object. Different environmental conditions are reflected in the grasp score, provided through a novel sensory pipeline, which is able to generate grasps for objects with unknown initial locations. To represent a distinct heterogeneity between the robots used in our task, different constraints were placed on the grippers for each robot. For the PR2, the enforced constraints only allow the robot to get grasps in which the gripper is sideways, i.e., nearly parallel to the floor. For the Baxter, a similar method is used to enforce the gripper to grasp the objects top down, i.e., the gripper is nearly perpendicular to the floor. These constraints enforce the maximum distinction in grasping functionality between the robots to illustrate the extent of heterogeneity for which the proposed method allows. These constraints force several of the objects to become nearly un-graspable by the PR2, namely, the apple, the orange, and the sandwich. The grasp scores for these objects returned by our novel perception-manipulation pipeline (Section 2.4.2) are close to 0 due to the fact that the objects are too wide for the PR2’s gripper to fit around them when the gripper is constrained in this manner. The constraints on the Baxter do not inhibit its grasp capabilities for any of the objects, but result in different grasp scores for the robot in different environments. These metrics are combined using a weighted linear combination, as shown in Equation 1:

$$\begin{array}{c} activation\text{\_}potential=w_{d}\cdot distance\text{\_}score+w_{g}\cdot grasp\text{\_}score \end{array}$$ (1)

$w_{d}$ and $w_{g}$ represent weights assigned to each metric, distance_score encapsulates how far the end effector is from the object to be grasped, and grasp_score encodes how good is the grasp returned from the grasp pipeline. Details on the grasp_score are provided in Section 2.4.2. The distance_score is computed in Equation 2, where ${\vec{x}}_{\mathrm{obj}}$ and ${\vec{x}}_{\mathrm{arm}}$ represent the 3D positions of the object and arm, respectively.

$$distance\text{\_}score=\frac{1}{\left\|{\vec{x}}_{\mathrm{obj}}-{\vec{x}}_{\mathrm{arm}}\right\|}$$ (2)

The values of weights used can be selected to assign a higher or lower significance to each metric; in this work $w_{d}=1$ and $w_{g}=0.001$ , to ensure that the grasp score is the same order of magnitude as the distance metric.

This metric is incorporated into our distributed control architecture through the activation potential (Section 2.2). The activation potential for each individual pick and place node is computed and updated at each step using Equation 1, which takes into account the different metrics described above. This value is then used by each robot to determine what behavior node to activate. In order to ensure proper coordination with the other robot teammates, such that no two robots decide to execute the same behavior, the process described in Section 2.2 is followed. Incorporation of the metric allows the task allocation to account for the robot’s degree of ability to perform a task; the metric 1) covers a discrete and continuous spectrum and 2) is variable in different environmental conditions.

#### 2.4.2 Perception-manipulation pipeline

**FIGURE 9:**
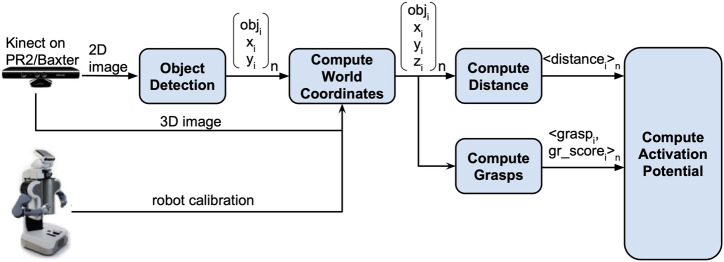
Perception-manipulation pipeline.

The metrics used in Equation 1 are continuously computed from sensory data, through the pipeline shown in Figure 9. This pipeline is capable of generating grasps for objects with unknown initial locations. This allows for two contributions to the previously developed architecture described in Section 2.2: 1) the metric is able to accurately reflect the varying capabilities of the robots in different environmental conditions and 2) the architecture is able to automatically grasp objects. In the previous architecture, the grasps of the objects were pre-determined based on specific orientations. Utilizing this pipeline, grasps can be automatically generated for objects with arbitrary positions and orientations. This enables us to extend the architecture to allow for dynamic task allocation with different environmental conditions.

The perception-manipulation pipeline consists of multiple modules. The first module performs object detection using our previously developed vision system described in (Hoseini et al., 2019). This system uses input from a Kinect mounted on the robot’s head to detect the objects.

The object detection module returns objects and their locations in the 2D camera view; this is combined with depth information to obtain locations of the objects in the robot’s 3D coordinate frame. This is used to compute the end effector distance to each of the objects in the scene, and as input to a module that computes possible grasps for the detected objects, based on the GPD library (Gualtieri et al., 2016). Given a point cloud, the GPD library is designed to return a set of grasps (6-DOF position and orientation, grasp quality score). According to (Gualtieri et al., 2016), the grasps returned by GPD are robust and reliable in cluttered environments (grasps were shown to have a 93% success rate).

However, GPD makes one of two assumptions: 1) any graspable object in the scene is acceptable; or 2) only a single object is in the point cloud. These assumptions did not hold for our use, so we made several modifications to utilize GPD. To force GPD to return grasps on a single object, each object in the task tree utilizes its own instance of the GPD library, which observes a subset of the point cloud from the Kinect centered around the location of the object (from the object detection module). As the object moves around, its respective location in the point cloud changes. The GPD workspace associated with that object has to be continually updated as well, which required some modifications to GPD’s interface with the Robot Operating System (ROS) (Quigley et al., 2009). We modified GPD further to extract, for each object, the grasp with the highest rating score from the set of possible grasps.

These modifications to GPD resulted in a single rating score per object. We take the continuous-valued score returned by GPD and add two additional discrete filters to the score in order to obtain the final grasp score. The first filter checks whether or not the grasp is within the robot’s reachable space. If it is, the original score is kept. Otherwise, the grasp score is set to 0. The grasp score returned by the first filter is then fed into the second filter. The second filter checks whether the orientation of the grasp is within a provided range of orientations which represent the possible configurations of the robot’s gripper. For the purposes of this experiment, this range is hard-coded to enforce different grasp patterns on the robots as specified in Section 2.4.1 but could be automatically computed using the computed grasp position and an inverse kinematics solver for the robot. If the grasp is within the set of feasible configurations for the robot, the score passed from the first filter is kept, otherwise the score is set to 0. The result of this second filter becomes the final grasp score for the given object. This grasp score therefore provides a measure of the robot’s perceived capability for grasping an object in varying environmental conditions with unknown orientations.

The grasps generated by GPD along with their newly computed grasp scores are returned by the compute grasps module. The distance and the grasp scores are then used to compute the *activation potential* for each of the objects in the scene as explained in Equation 1. This value is used by the update loop to determine which node should be activated by each robot, as described in Section 2.2.

## 3 Results

### 3.1 Experimental setup

The proposed architecture has been validated in two types of experiments, specifically designed to illustrate the key proposed contribution: 1) the ability to coordinate complex task execution in teams consisting of both human and multiple robot teammates and 2) the ability to handle dynamically changing capabilities in the context of heterogeneous multi-robot teams.

The objects used in the experiments include a wooden tea-set (consisting of a cup, a sugar container, and a teapot) in addition to several fake food objects (namely, an apple, a burger, an orange, and a sandwich). The task structures are shown in Figure 4, for the human-robots experiments, and in Figure 2 for the multi-robot experiments.

The human-robots task consists of placing some of the food/household objects in the order shown by the hierarchical tree representation: first, the burger and the cup have to be placed in their desired destinations, without any constraint on which is placed first, and then the teapot and sugar are placed at their destinations, also without any constraints.

The joint task structure for the multi-robot experiments consists of two main sub-tasks that can be executed in parallel. The first is a tea-setting task which is shown in the left sub-tree of the task structure. This sub task consists of first placing the cup, then placing the sugar and the teapot (in any order) to their goal locations. The second sub-task is the food-setting task which is shown in the right sub-tree of the task structure. This sub-task consists of first placing the sandwich, followed by placing either the orange or the apple, and then finally placing the burger to their respective goal locations.

The pick and place nodes take as input the desired grasp location of an object provided by the perception-manipulation pipeline (Section 2.4.2) and place the object at a pre-specified location. End-effector trajectories to the grasp location are generated using MoveIt (Sucan and Chitta, 2013). The right arm on each robot was used. The complete motion must be completed in order for the pick and place node to be marked as done. Until the place command finishes, the robot waits before it activates another node, since only one node per robot can be doing work at any given time.

**FIGURE 10:**
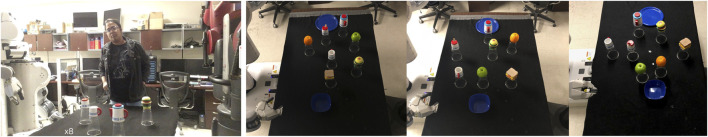
Experimental setup. Left: Human and 2 robots scenario. Right: placement of objects for the three distinct robot-robot scenarios. Figure contains images of the author(s) only.

The initial setups for the human-robot and multi-robot experiments are shown in Figure 10. For this setup, the Baxter and PR2 robots were placed on either side of a table with the objects in between them. In the human-robot experiments, the human sits at one of the free sides of the table. The goal locations for the objects are as follows: the burger and the teapot are placed on the human side of the table, while the cup and the sugar are placed on the opposite side.

For the multi-robot experiments, three different scenarios were performed, in which the objects were placed in different locations to show that the architecture can dynamically determine different task allocations based on the specifics of the environment. Figure 10 shows the placement of the objects for the different scenarios. For all of the scenarios, the goal locations were as follows: the apple and the orange are placed into the bowl on the right side of the PR2; the cup, sugar, and teapot are placed next to the bowl; and the burger and the sandwich are placed onto the plate on the left side of the PR2. In order to assess the impact of the proposed metric as defined in Section 2.4.1, each scenario consisted of two separate trials. The first trial used a metric (named distance-only) that took into consideration only the distance from the robot’s gripper to the objects for the activation potential (first term in Equation 1). The second trial used the full heterogeneity metric (named distance-and-grasp) shown in Equation 1 which incorporates both the distance and the grasp score into the activation potential.

### 3.2 Experimental results

#### 3.2.1 Human-robot experiments

**FIGURE 11:**
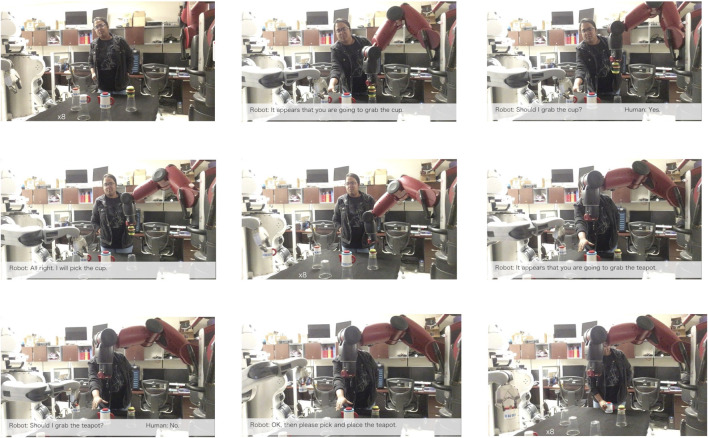
Key stages of the human-robot experiment. Figure contains images of the author(s) only.

Figure 11 illustrates key stages from the execution of the experiments performed with the team of 2 humanoid robots and a human teammate. Stage 1 shows the initial setup, prior to the start of the experiment. In stage 2, the Baxter robot selects the task of placing the burger, while the PR2 and the human both sel ect to place the cup. Through the decision process described in Section 2.3, the robot detects this conflict and initiates a dialog as shown in Stage 3. Through the dialog, the human acknowledges giving up on the task and allowing the robot to continue. Stage 4 the robot confirms receiving the human’s decision and activates the cup placing task. In stage 5 both robots execute their current tasks (place-cup by the Baxter and place-burger by the PR2). At this time, two sub-tasks remain to be executed, which are placing the sugar and the teapot. Due to closeness to the sugar cup, the PR2 robot decides to place it, with no other teammates being interested in that task (stage 6). At the same time, the Baxter and the human both decide to place the teapot. The Baxter robot initiates a dialog and gets confirmation from the user that they want to continue with that task (stage 7). The Baxter acknowledges the decision of the human teammate and lowers the activation for placing the teapot (stage 8). Stage 9 shows the final step of the task being executed by the human teammate.

#### 3.2.2 Heterogeneous robot team experiments

**FIGURE 12:**
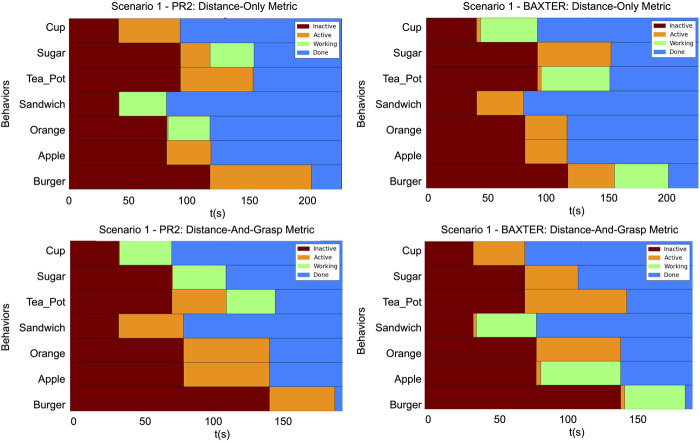
The timing diagrams for Scenario 1. These diagrams represent the times at which the state of a node in a given task tree changed. Top row: Provides the timings for the PR2 and the Baxter using the distance-only metric. Bottom row: Provides the timings for the PR2 and the Baxter with the distance-and-grasp metric which utilizes the heterogeneity component. Within each graph: Each row corresponds to a behavior node named according to its corresponding object. The horizontal axis is increasing time. Brown → *inactive*, Orange → *active*, Green → *working*, and Blue → *done*.

**FIGURE 13:**
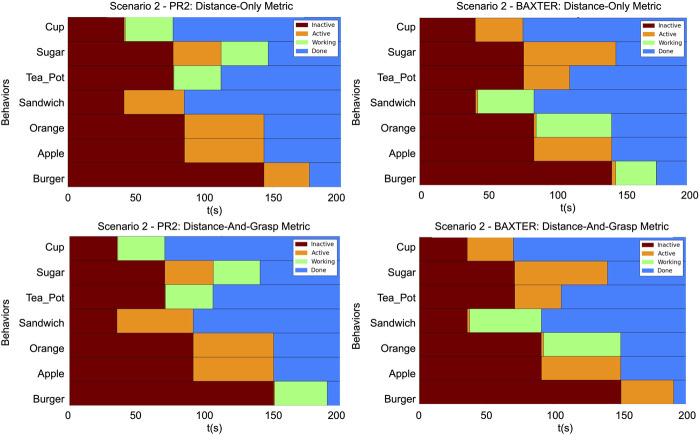
The timing diagrams for Scenario 2. These diagrams represent the times at which the state of a node in a given task tree changed. Top row: Provides the timings for the PR2 and the Baxter using the distance-only metric. Bottom row: Provides the timings for the PR2 and the Baxter with the distance-and-grasp metric which utilizes the heterogeneity component. Within each graph: Each row corresponds to a behavior node named according to its corresponding object. The horizontal axis is increasing time. Brown → *inactive*, Orange → *active*, Green → *working*, and Blue → *done*.

**FIGURE 14:**
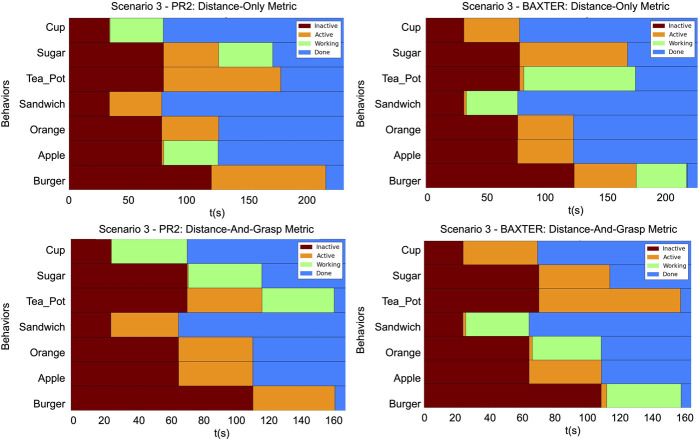
The timing diagrams for Scenario 3. These diagrams represent the times at which the state of a node in a given task tree changed. Top row: Provides the timings for the PR2 and the Baxter using the distance-only metric. Bottom row: Provides the timings for the PR2 and the Baxter with the distance-and-grasp metric which utilizes the heterogeneity component. Within each graph: Each row corresponds to a behavior node named according to its corresponding object. The horizontal axis is increasing time. Brown → *inactive*, Orange → *active*, Green → *working*, and Blue → *done*.

The timing diagrams for the different scenarios are shown in Figures 12–14. In each figure, the results of the two trials within a single scenario are shown. The top row illustrates the results of the first trial using the distance-only metric on the PR2 and Baxter. The bottom row illustrates the results of the second trial using the distance-and-grasp metric. Each of the individual timing diagrams illustrate the change of state of each node in the task tree for a given robot. The different color bars in the figure represent the times during which a particular pick and place behavior node is in one of the following states: inactive, active, running, or done. The intervals corresponding to the running state identify when a given node is being executed and are thus indicative of the order in which various sub-tasks have been performed. Additionally, the grasp scores for the different scenarios for each robot are given in Tables 1, 2. These scores differ across trials and robots due to the different environmental conditions in each scenario as well as different grasp capabilities of each robot. The results of each scenario are discussed below.

**TABLE 1:** Table of grasp scores for the PR2 for each scenario (rounded to nearest integer).

PR2	Scenario 1	Scenario 2	Scenario 3
Cup	**25**	**33**	**14**
Sugar	**57**	**17**	**23**
Tea_Pot	**13**	**41**	**50**
Sandwich	0	0	0
Orange	0	0	0
Apple	0	0	0
Burger	15	**91**	5

Scores in bold are the objects which the PR2 grabbed during each scenario.

**TABLE 2:** Table of grasp scores for the Baxter for each scenario (rounded to nearest integer).

Baxter	Scenario 1	Scenario 2	Scenario 3
Cup	10	8	2
Sugar	14	2	2
Tea_Pot	2	17	13
Sandwich	**44**	**26**	**27**
Orange	12	**14**	8
Apple	**13**	9	7
Burger	**16**	13	**11**

Scores in bold are the objects which the Baxter grabbed during each scenario.

In Scenario 1 there were several differences in the allocation of objects between the trials for the distance-only and the distance-and-grasp metrics. For the distance-only trial, first the PR2 picked up the sandwich while the Baxter grabbed the cup, then the PR2 grabbed the orange while the Baxter grabbed the teapot, and lastly the PR2 picked up the sugar and the Baxter grabbed the burger. Since the metric used in this trial only utilizes distance to the objects, this allocation grasps the closest objects first, while adhering to the constraints defined in the task structure. However, because the PR2 cannot accurately grasp the sandwich or the orange due to the constraints of the gripper, these objects get knocked over during the execution of this task. At this time, the robots do not have the capability to detect that the object was dropped, so they assume that the place behavior was successful and the task will continue on. This will be addressed in future work. In the trial utilizing the grasp score, the cup, sugar, and tea were all allocated to the PR2, and the other objects were allocated to the Baxter. This illustrates that by utilizing the grasp score in the metric for the activation potential, the architecture is able to allocate objects which are graspable by the robot, while still adhering to the various types of constraints provided in the task structure.

Scenario 2 illustrates the continuous-valued element of the proposed metric. At the time when the allocation of the burger was determined, the Baxter’s gripper was at the goal location for the orange and the PR2’s gripper was at the goal location for the sugar. Due to the fact that the pick location of the burger is slightly closer to the orange’s goal location than to the sugar’s goal location, in the original trial the Baxter picked up the burger since only the distance from gripper to object was used. The burger’s grasp score for the PR2 is higher than that of the Baxter (91 vs. 13). Thus, in the trial which utilizes the grasp score, the burger is allocated to the PR2 instead of the Baxter. This illustrates that the proposed metric is able to properly allocate objects based on a skill which can be performed to various degrees (continuous-valued score) rather than a simple binary (yes/no) skill.

Scenario 3 illustrates a combination of the findings from the previous two scenarios. Utilizing the proposed distance-and-grasp metric which accounts for the variable heterogeneity: 1) allows the objects to be allocated such that the robots can grasp all of the objects allocated to them and 2) is able to allocate objects with a higher chance at being grasped according to a continuous-valued metric. Using the distance-only metric, the apple is allocated to the PR2. However, the apple cannot be reliably grasped by the PR2 due to the gripper constraints. Thus, in the trial with the distance-and-grasp metric, the apple is instead allocated to the Baxter. This is similar to the finding in Scenario 1. In the distance-and-grasp metric trial, when the burger is allocated, the PR2 is at the sugar goal location and the Baxter is at the orange goal location. The PR2 has a higher grasp score on the teapot than the burger (50 vs. 5); while the Baxter’s scores are very similar (11 vs. 13). Thus, the PR2 grabs the teapot while the Baxter grabs the burger. This illustrates that, similar to Scenario 2, the distance-and-grasp metric which accounts for the heterogeneity is able to allocate the objects to the robots which have a higher chance of grasping them reliably.

These scenarios illustrate that the inclusion of the heterogeneity component in the scoring metric of the architecture results in allocations of the objects to the robots which are best suited to grasp them. The proposed architecture is able to handle variable heterogeneity during the task allocation which takes into account the most recent environmental conditions as the task progresses while adhering to the complex task constraints.

## 4 Discussion

### 4.1 Future work

An immediate extension of this work is to include additional features in the computation of the robot’s performance metric, such as a feature that provides insight into the efficiency of the trajectories computed to reach the objects and the destination. Additionally, the use of both arms for each robot can lead to increased team efficiency. Specific modules for obstacle avoidance will be developed to avoid collisions during the task. Furthermore, an additional capability will be added to track the status of the task execution in order to detect failed actions and then re-attempt them. Furthermore, to add to the repertoire of current experiments, new scenarios will be developed that would rely not only on the robot’s manipulation capabilities, but also on their navigation skills to reflect further heterogeneity between robots, since the PR2 robot is mobile but the Baxter is not.

### 4.2 Conclusion

This paper presents a real-time distributed control architecture for collaborative task execution of manipulation tasks by heterogeneous human-robot teams. The main contributions of the approach are the ability to coordinate task execution with robots and human teammates, the ability to handle variable robot heterogeneity, the ability to handle automatic grasping of objects with unknown initial locations, and the collaborative execution of tasks with hierarchical representations and multiple types of constraints. This is achieved through a theory of mind approach in which robots store simulated mental models of the human’s task and through the use of a continuous-valued metric that encodes a robot’s ability to perform a particular task component; the metric is updated continuously during task execution, allowing for dynamic task allocation that takes into account the most recent environmental conditions.

Additionally, the architecture provides a novel perception-manipulation pipeline which is able to automatically generate grasps on objects with arbitrary positions and orientations. This pipeline is utilized by the updated metric which allows it to accurately reflect the varying capabilities of the robots in different environmental conditions. Experimental validation is performed with teams of two humanoid robots and a human, as well as two heterogeneous humanoid robots performing household manipulation tasks. The outcomes of the experiments support the proposed contributions. First, humans and robots can effectively coordinate their actions and correctly execute tasks with complex constraints. Second, different environmental conditions result in different and continuously changing values for the robot’s task execution ability, resulting in dynamic task allocation among the heterogeneous robot team performing complex hierarchical tasks.

## Data Availability

The original contributions presented in the study are included in the article/Supplementary Material, further inquiries can be directed to the corresponding author.
