# Supplementary figures and images for: Simulation theory of mind for heterogeneous human-robot teams
Source: Front Robot AI. 2025 Jun 17;12:1533054. doi: 10.3389/frobt.2025.1533054 (PMC12209716; doi:10.3389/frobt.2025.1533054)

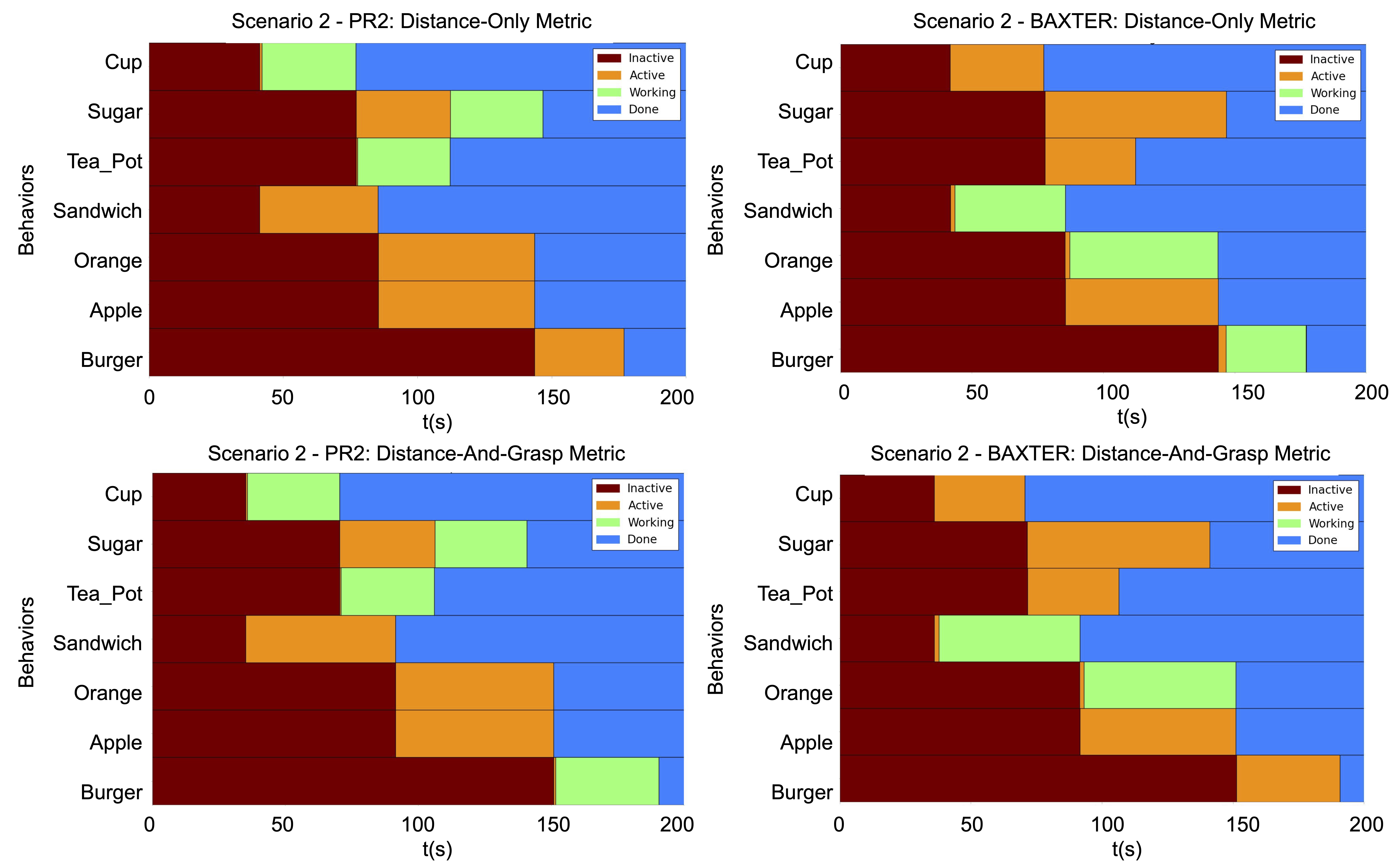

Supplement: Supplementary file 1 [file Image1.JPEG]

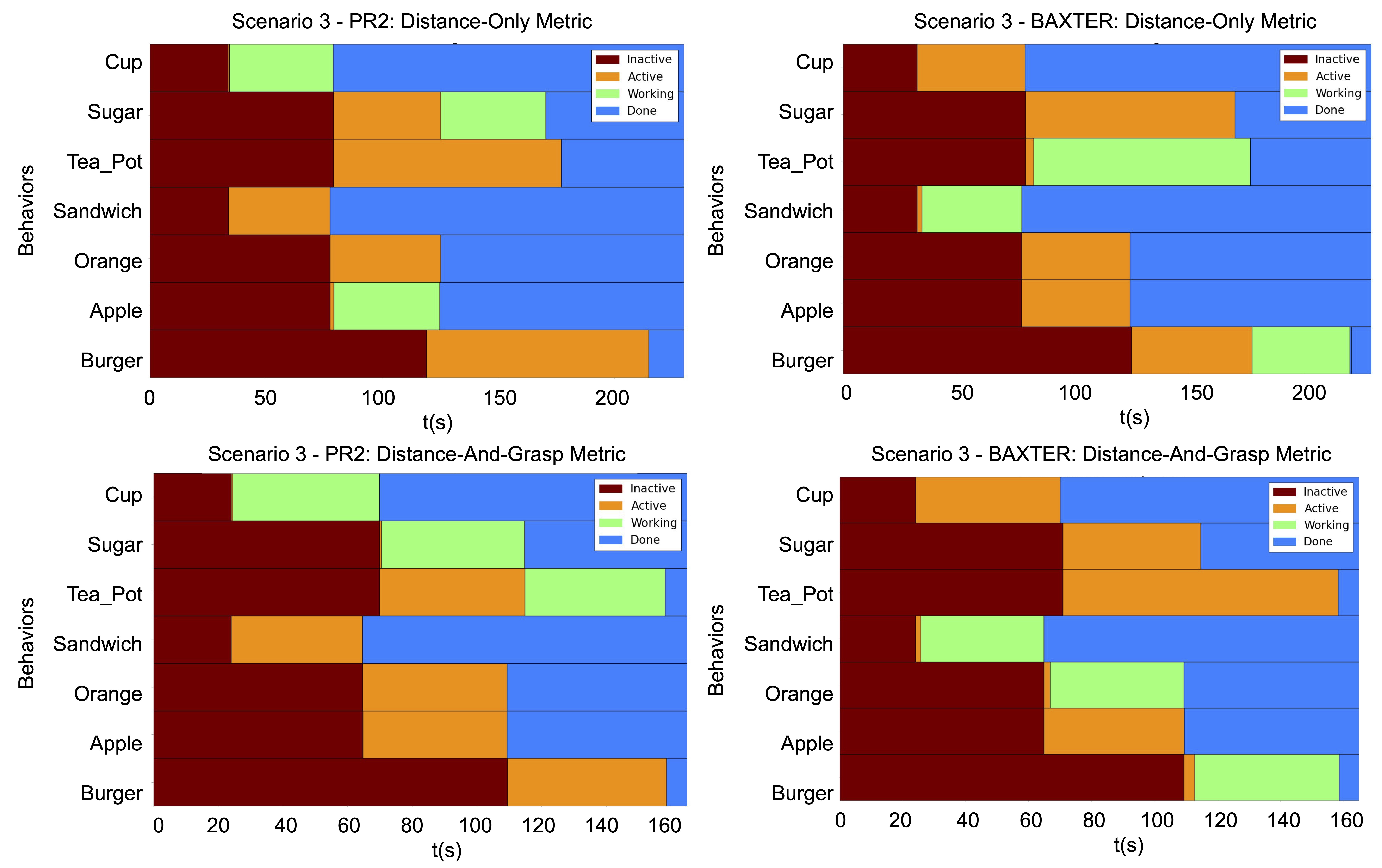

Supplement: Supplementary file 2 [file Image2.JPEG]
